# IL-6/STAT3 Induced Neuron Apoptosis in Hypoxia by Downregulating ATF6 Expression

**DOI:** 10.3389/fphys.2021.729925

**Published:** 2021-10-21

**Authors:** Simin Zhou, Zhifeng Zhong, Pei Huang, Bin Xiang, Xiaoxu Li, Huaping Dong, Gang Zhang, Yu Wu, Peng Li

**Affiliations:** ^1^Department of High Altitude Operational Medicine, College of High Altitude Military Medicine, Army Medical University, Chongqing, China; ^2^Key Laboratory of Extreme Environmental Medicine, Ministry of Education of China, Army Medical University, Chongqing, China; ^3^Key Laboratory of High Altitude Medicine, Army Medical University, Chongqing, China; ^4^Department of High Altitude Physiology and Pathology, College of High Altitude Military Medicine, Army Medical University, Chongqing, China

**Keywords:** hypoxia, cognitive impairment, STAT3, ATF6, ER stress

## Abstract

**Background:** Neuron apoptosis, regulated by endoplasmic reticulum (ER) stress in the hippocampus, is an essential factor influencing the cognitive impairment induced by hypobaric hypoxia. Hypoxia mainly changes the activating transcription factor (ATF6) pathway of ER stress. However, the role of ATF6 in neuron survival, apoptosis, and upstream regulation is still controversial.

**Methods:** We established a hypobaric hypoxia-induced C57BL/6 murine model and cell lines exposed to 1% hypoxia, including PC12 and HT22. First, we tested the expressions of interleukin 6 (IL-6), IL-1β, and IL-10 in C57BL/6 mice’s hippocampus under hypoxia using enzyme-linked immunosorbent assay (ELISA). We determined the signal transducer and activator of transcription 3 (STAT3) phosphorylation at tyrosine (Tyr)705 by western blot and the expression of ATF6, 78-kDa glucose-regulated protein (GRP78), and C/-EBP homologous protein (CHOP) related to ER stress by immunofluorescence (IF), western blot, and qRT-PCR; they were then verified on the cell model. Additionally, IL-6 (40 ng/mL) and STAT3 siRNA were used to treat the PC12 cells for 48 and 4 h to activate or silence STAT3, respectively. Subsequently, the cells of siRNA group were exposed to 1% hypoxia for 48 h. Furthermore, the ATF6 and CHOP expressions were detected with western blot and qRT-PCR. Finally, we examined the binding of STAT3 to the ATF6 promoter by chromatin immunoprecipitation (ChIP)-seq.

**Results:** The results showed that IL-6 increased, IL-10 decreased in the hypoxia group, and IL-1β showed no difference between the hypoxia and the normoxia groups. Neuron apoptosis was significantly elevated by exposure to hypoxia for 48h in PC12 cells. The hypobaric hypoxia-induced ER stress proteins, ATF6, GRP78, and CHOP, and the p-STAT3 (Tyr705) expressions increased both in *in vivo* and *in vitro*. Besides, STAT3 silencing significantly promoted the ATF6 expression and inhibited CHOP, while STAT3 activation downregulated the expression of ATF6 and upregulated CHOP in PC12 cells. The ChIP-seq assay demonstrated that p-STAT3 (Tyr705) protein could bind to the ATF6 promoter region in HT22 cells.

**Conclusion:** Phosphorylation of STAT3 at the Tyr705 site contributes to hypoxia-induced neuron apoptosis by downregulating ATF6, which might explain the inflammatory reaction and apoptosis of the hippocampal neurons induced by ER stress.

## Introduction

Hypoxia encountered causes cognitive impairment. Cognitive functions, such as memory and learning, are a crucial nature of the hippocampus. It has been verified that hypoxia-induced neuronal death is one of the significant factors bringing about cognitive impairment (Chen et al., 2017; Ding et al., 2018; Ji et al., 2021).

We studied the possible latent mechanism of hypoxia-induced cognitive injury. Hippocampal neuron apoptosis causes cognitive decline (Li et al., 2002), including high-altitude hypoxia and intermittent hypoxia (Nyakas et al., 1996; Li et al., 2012; Shi et al., 2018). The hypoxia-induced neuron apoptosis (Lopez-Hernandez et al., 2015), resulting in cognitive dysfunction, is associated with microglial activation, autophagy, endoplasmic reticulum (ER) stress, and so forth (Lopez-Hernandez et al., 2015; Xie et al., 2016; Liu et al., 2017; Shi et al., 2018). Inflammatory factors, such as interleukin 6 (IL-6), activate ER stress (Shin et al., 2012; O'Neill et al., 2013) that leads to neuron death in the central nervous system (CNS; Stefani et al., 2012; Roussel et al., 2013). There is increasing evidence indicate that ER stress plays an important role in cells apoptosis induced by ischemia/hypoxia *in vitro* (Chen et al., 2008; Roussel et al., 2013) and *in vivo* (Galehdar et al., 2010; Lopez-Hernandez et al., 2015).

The unfolded protein response (UPR) in the lumen of ER is activated by the storage of unfolded and/or misfolded proteins. As a sophisticated and harmonized flexibility mechanism, the UPR reestablishes the equilibrium of ER functions. The stress sensors of ER, ATF6, inositol requiring enzyme1 (IRE1), and PKR-like eIF2a kinase (PERK) check the storage of the unfolded and/or misfolded proteins at the beginning of the ER stress and start the UPR. However, some studies confirmed ATF6 activation rather than IRE1 and PERK activation in the primary neuronal cells and the adherent sub-clone of rat pheochromocytoma (PC12) cells, a common neuron-like model, after exposure to hypoxia (Gharibani et al., 2013; Liu et al., 2015; Prentice et al., 2017). ATF6 is an ER-resident transmembrane protein. After ATF6 activation, adjusted proteolysis was used to make the cytoplasmic domains rift from the membrane anchor. Being an active transcription factor, the rifted N-terminal fragment transfers to the karyon and raises the expression of gene-encoding proteins that strengthen the ER protein-folding capacity. ATF6 is a neural branch that protects UPR; ATF6 reduction is inclined to touch off apoptosis along with activated proapoptotic IRE1α and PERK subfields of UPR (DeGracia et al., 2002; Mercado et al., 2016). However, a study reported taurine’s protective effects in neurons against hypoxia, reoxygenation by inhibiting p-IRE-1 or ATF6 rather than the pathway of PERK (Prentice et al., 2017). Presently, the role of ATF6 in the mediated neuron-cell survival or apoptosis is still controversial. The upstream mechanism of ATF6-mediated cell survival and apoptosis remains unknown.

The ER stress may be induced by an inflammatory response through several pathways, like IL-1β, IL-6, and TNFα. They are pro-inflammation cytokines. IL-6 is well known for its pro-inflammation effects. However, it also has anti-inflammatory, regenerative, and pro-resolution properties (McElvaney et al., 2021). IL-6 binds to its receptor thereby activating Janus kinase (JAK), which in turn activates signal transducer and activator of transcription 3 (STAT3) by tyrosine (Tyr705) phosphorylation (Wrighting and Andrews, 2006). STAT3 is a transcription factor and an intracellular signal sensor activated by growth factors, cytokines, and tyrosine kinases (Darnell, 1997; Levy and Lee, 2002; Hillmer et al., 2016). Hypoxia induces STAT3 activation by Tyr705 phosphorylation, shuttles from the cytoplasm to the karyon, discerns STAT3-specific DNA motifs, and expresses target genes (Groner et al., 2008; Greenhill et al., 2011; Gao et al., 2015). Previous studies have shown that STAT3 gets activated after exposure to hypoxia (D’Angelo et al., 2016; Hristova et al., 2016). Our previous research found that chronic hypoxia promoted the GRP78 and ATF6 expressions through the STAT3 pathway activation to protect the cardiomyocytes against ER stress-related apoptosis (Zhou et al., 2019). Furthermore, STAT3 activation has been observed in neuropathological conditions comprising adult ischemia (Justicia et al., 2000) and neonatal hypoxic-ischemic (HI) injury in the brain (Shrivastava et al., 2013). The STAT3 signaling pathway involvement has also been reported in the brain HI injury (D’Angelo et al., 2016; Hristova et al., 2016). Neuronal STAT3 functions in the pathway, causing potential apoptosis and HI injury. However, the downstream mechanism of STAT3 in neuron death is not yet clear.

This study found that hypoxia promoted hippocampal inflammatory response and ER stress in C57BL/6 mice and PC12 cells and caused neuron injury. We also evaluated the injury effect of STAT3 on neurons under hypoxic conditions and for the first time explored the regulatory effects of STAT3 on ATF6 in neurons. The study will enrich our knowledge on hypoxia-induced hippocampal neuron apoptosis.

## Materials and Methods

### Mice Treatment

Third Military Medical University, an experimental animal center, provided 8–10weeks (18±2g) C57BL/6 male mice. They were kept at 25°C, with 45% humidity, and exposed to a 12-h light −12-h dark cycle. The hypoxia group was exposed to an altitude of 6,000m for 7days. The normoxic control group was kept at the normal atmosphere of about 300m for 7days. All procedures followed the standards of experimental animal ethics.

After the hypoxia treatment, a small amount of chloral hydrate (10%, w/v) was used to inject the mice to narcosis, which were then perfused with 400mL of normal saline (0.9%, v/v) intracardially; the hippocampus was harvested for western blot and quantitative real-time PCR (qRT-PCR). This was followed by perfusing with 400mL of normal saline (0.9%, v/v) and 350mL of paraformaldehyde (4%, v/v). Finally, we decapitated the mice and fixed their brains in paraformaldehyde (4%, v/v) overnight. The brains were dewatered using 30% sucrose in the same solution; sections of brain serial coronal (30μm) were collected with a cryostat (CM1900, Leica Microsystems, Germany) at −20°C and used further for immunofluorescence (IF).

### Cell Culture

The cell repository of the Chinese Academy of Sciences offered the PC12 cell line (high differentiation). The mouse hippocampal neurons HT22 cell line was purchased from iCell (Shanghai, China). We cultured PC12 cells in the RPMI-1640 medium (HyClone) and HT22 cells in the DMEM high glucose medium (Gibco), supplemented with 10% (v/v) fetal bovine serum (FBS; Gibco) and 1% (v/v) penicillin-streptomycin solution at a temperature of 37°C plus 5% CO_2_.

For the experiments, the cells were placed in a hypoxic chamber with 1% O_2_, 5% CO_2_, and 94% N_2_ for the design time. The normoxic control group incubated cells for the same duration with 5% CO_2_ and 95% air.

### Apoptosis Analysis by Flow Cytometry

Annexin V-FITC/PI double-staining and flow cytometry analyses were utilized to detect the apoptotic cells. Briefly, the PC12 cells were subjected to incubation at 1% oxygen concentration for 24 or 48h, and the cells were harvested and resuspended in phosphate-buffered saline (PBS) at 1×10^5^cells/mL. The PC12 apoptosis was detected using the Annexin V staining Protocols assay kit (Affymetrix eBioscience, 88-8005-72, United States) based on the manufacturer’s protocol. We added 5μL of annexin V-FITC and 195μL of annexin V-FITC binding buffer into the mixture after a 5-min centrifugation at 500×*g* at a temperature of 25°C. The solution was gently vortex-mixed, and the mixture was incubated for 10min in the dark at room temperature. Finally, after another centrifugation, 10μL PI and 190μL of FITC-conjugated annexin V binding buffer were supplemented. The samples were lightly vortex-mixed and assayed with a FACSCalibur Flow Cytometer (Becton Dickinson, San Jose, CA, United States) within half an hour.

### Cell Viability

The CCK-8 assay was carried out as reported in previous literature (Heng et al., 2010). The PC12 and HT22 cells were incubated in an oxygen-deficient chamber for 0, 24, or 48h and rinsed twice with PBS before 10μL of CCK-8 solution (CK04, Dojindo Laboratories, Japan) and 100μL of the substrate were added to it. The absorbance was detected at 450nm by a Multiskan Go Microplate Absorbance Reader (Thermo, Massachusetts, United States) by incubating the microplate at a temperature of 37°C for 2h. Cell viability was defined as the proportion of the viable cells compared to the control cells (100%). We repeated all experiments thrice, and the results were expressed as mean±SD.

### ELISA

The cytokines (IL-6, IL-1β, and IL-10), which contributed to inflammation, were assessed using tetramethylbenzidine (TMB) ELISA kit (M6000B, MLB00C, M1000B, R&D, United States) based on the manufacturer’s protocol. A microplate reader (Thermo, Massachusetts, United States) was utilized to detect the absorbance at 450nm.

### Immunofluorescence

The samples were blocked by QuickBlock™ blocking buffer for 1h (Beyotime Biotech Inc., P0260, Shanghai, China) for IF staining. The brain sections were afterward subjected to incubation with rabbit anti-GRP78/BIP (1:500, Proteintech, 66,574-1-Ig, United States) or rabbit anti-CHOP (1:500, Proteintech, 15,204-1-AP, United States) overnight at 4°C. Cy3-labeled Goat Anti-Rabbit IgG (H+L; 1:200, Beyotime Biotech Inc., A0516, Shanghai, China) was used to visualize the binding, while the antifade mounting medium with DAPI (Beyotime Biotech Inc., P0131, Shanghai, China) was applied to stain the nuclei. We imaged the immunolabeled brain slices with a CLSM (IX81, Olympus, Tokyo, Japan) and then analyzed them with a three-dimensional (3D) constructor, ImageJ software (National Institutes of Health, Bethesda, MD, United States).

The PC12 cells were subjected to incubation in rabbit anti-GRP78/BIP (1:500, Proteintech, 66574-1-Ig, United States) or rabbit anti-ATF6 (1:500, Proteintech, 66563-1-Ig, United States) or rabbit anti-CHOP (1:500, Proteintech, 15204-1-AP, United States) overnight at a temperature of 4°C. The other steps were the same as the brain sections.

### Knockdown With siRNA

The PC12 cells were transfected siRNA targeted STAT3 to knockdown the STAT3 expression. Briefly, to prepare the lipid–siRNA complex, siRNA (GenePharma; Table 1) and Lipofectamine 2000 (Invitrogen) were diluted in Opti-MEM (Invitrogen) for 5min. Next, we mixed siRNA and Lipofectamine 2000 solution and incubated them for an extra 20min. After gently layering the siRNA-Lipofectamine 2000 complex, we placed it in the cell cultures for 4h. Finally, the substrate was replaced completely. The cells were thus transfected and used for further processes.

**Table 1:** Oligonucleotide sequences used in this study.

Primer name	Sequences	Purposes
STAT3-siRNA-2106	5'-CCAGUCUGUAGAACCAUAUTTAUAUGGUUCUACAGACUGGTT-3'	WB、PCR
STAT3-FP	5'-TGGGTCTGGCTAGACAAT-3'	PCR
STAT3-RP	5'-CGTTGGTGTCACACAGAT-3'	PCR
ATF6-FP	5'-CCAGCAGAAAACCCGCATTC-3'	PCR
ATF6-RP	5'-AACTTCCAGGCGAAGCGTAA-3'	PCR
GRP78-FP	5'-AACCCAGATGAGGCTGTAGCA-3'	PCR
GRP78-RP	5'-ACATCAAGCAGAACCAGGTCAC-3'	PCR
CHOP-FP	5'-GGAAGTGCATCTTCATACACCACC-3'	PCR
CHOP-RP	5'-TGACTGGAATCTGGAGAGAGCGAGGGC-3'	PCR

### Western Blot

The cells were rinsed twice with ice-cold PBS, and the cell lysates were extracted using the radioimmunoprecipitation (RIPA) lysis buffer (Beyotime Biotech Inc. P0013B, Shanghai China) containing 1mM PMSF. The BCA Protein Assay Kit (Beyotime Biotech Inc., P0009, Shanghai, China) was used to determine the lysate’s protein concentration. Equivalent amounts of protein from each sample were extracted using 10% sodium dodecyl sulphate-polyacrylamide gel electrophoresis (SDS-PAGE) and then transferred to a polyvinylidene difluoride (PVDF) membrane. The membrane, after blocking with 5% milk for 1h, was incubated with the primary antibody in the blocking buffer overnight at 4°C. The membrane was then rinsed three times with the blocking buffer and supplemented with the Goat Anti-Rabbit IgG(H+L) secondary antibody (Beyotime Biotech Inc., A0208, Shanghai, China) labeled with horseradish peroxidase (HRP) at a 1:2000 dilution in blocking buffer, and the membrane was then incubated for 1h at room temperature. Enhanced chemiluminescence (ECL) detection kit (BG0001, Bioground, Chongqing, China) was adopted to visualize antibody binding, and the images were captured using the Quality One software (Bio-Rad) scanner.

The primary antibodies and dilutions were as follows: anti-GRP78/BIP (1:200, Abcam, ab108615, United States), anti-ATF6 antibody (1:1000, Santa Cruz Biotechnology, SC-22799, United States), anti-GADD153/CHOP antibody (1:1000, Santa Cruz Biotechnology, SC-575, United States), anti-Phospho-STAT3 (Tyr705) antibody (1:1000, Cell Signaling Technology, 9145, United States), and anti-β-actin antibody (1:2000, BestBio, BBA1107-2, Shanghai, China).

### RNA Isolation and Quantitative Real-Time PCR

We extracted total RNA with RNAprep pure Micro Kit (Tiangen, DP420, Beijing, China); it was reverse transcribed to cDNA by the RNA PCR Kit (Takara, RR036A, Shiga, Japan) based on the manufacturer’s guidance. Subsequently, we amplified the cDNA product with the PCR amplification kit (Takara, RR820A, Shiga, Japan). Taking the β-actin gene as an internal control, we determined the relative gene expression with the comparative CT method. Table 1 illustrates the primer sequences. The PCR settings were set below: 95°C for 30s, 40cycles at 95°C for 5s, and 60°C for 30s. The 2^−∆∆Ct^ methods were utilized to calculate the relative expression fold change of mRNAs.

### Chromatin Immunoprecipitation Assay

The chromatin immunoprecipitation (ChIP) assay was done on HT22 cells cultured in IL-6 (40ng/mL) to promote the STAT3 activation by SeqHealth (Wuhan, China). Following the above-mentioned processes, the cell protein-DNA complexes were cross-linked using 1% formalin in PBS on a rocking device at room temperature for 15min. The formaldehyde was quenched by adding 125mM glycine and rocked for 5min at room temperature. Afterward, the cells were collected and treated with lysis buffer in 500μL per 5×10^6^ cells on ice for 10min. The chromatin was sheared to an average length of about 1kb using sonication. After ultracentrifugation (10min) with a refrigerator at 12,000×g, we collected the supernatants and performed ChIP using the EZ-Magna ChIP A/G Assay kit (Millipore, 17-10086, United States). The chromatin mixture was immunoprecipitated with anti-phospho-STAT3 (Tyr705) antibody (1:100, Cell Signaling Technology, 9,145, United States). The DNA was purified and sequenced with ChIP-seq.

### Statistical Analysis

Results are represented as mean±standard deviation (SD). The SPSS 18.0 statistical software program was used to make statistical analyses. The unpaired *t* test was utilized to compare two groups’ data, while we compared data from 3 or more groups with ANOVA. The differences were considered significant at *p*<0.05.

## Results

### Hypoxia Causes Inflammatory Response in Murine Hippocampus

**Figure 1:**
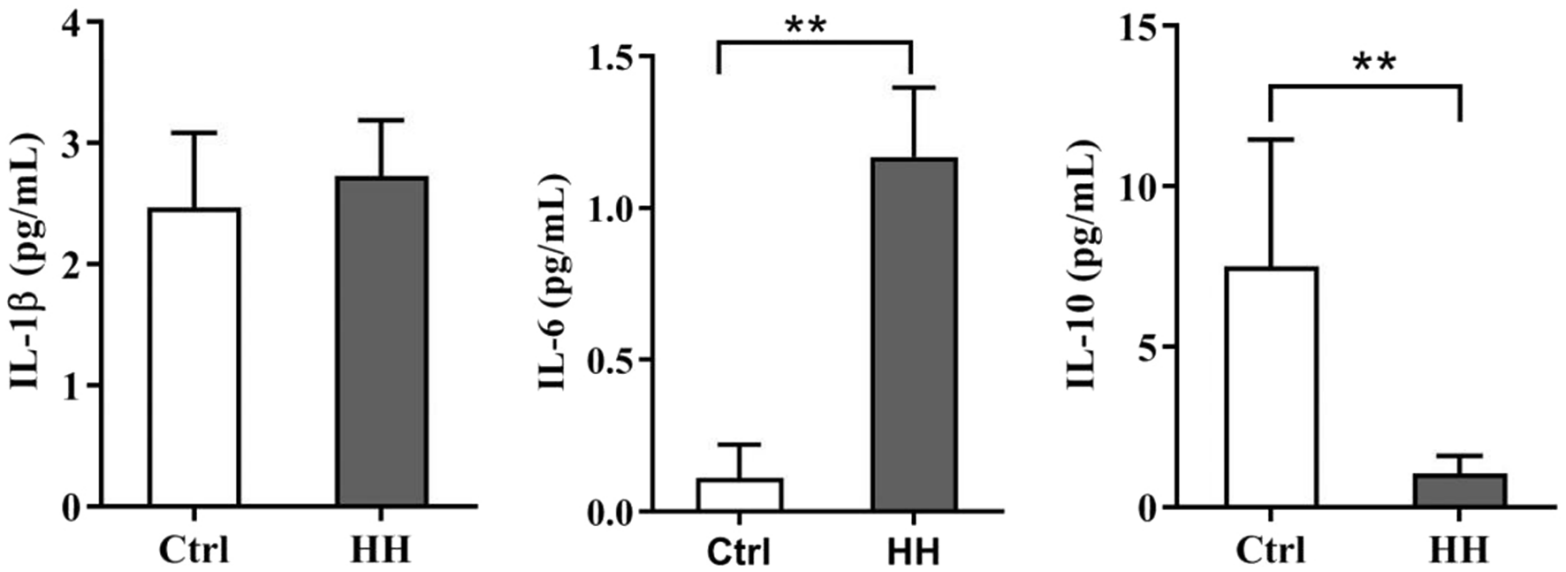
Hypobaric hypoxia (HH) exposure led to the inflammatory reaction in the hippocampus of C57BL/6 mice. Exposed to HH for 7days, the level of IL-1β, IL-6, and IL-10 was determined by ELISA. Data are presented as mean±SD (*n*=3). ^*^p<0.05, ^**^p<0.01 compared with the control group.

The inflammatory factors were observed to determine whether the hypobaric hypoxia-induced ER stress is related to inflammation in the hippocampus. The ELISA assay displayed a remarkable increase in IL-6, a dramatic drop in IL-10 in the hypoxia group, and no difference was seen in IL-1β between the hypoxia and normoxia groups (Figure 1).

**Figure 2:**
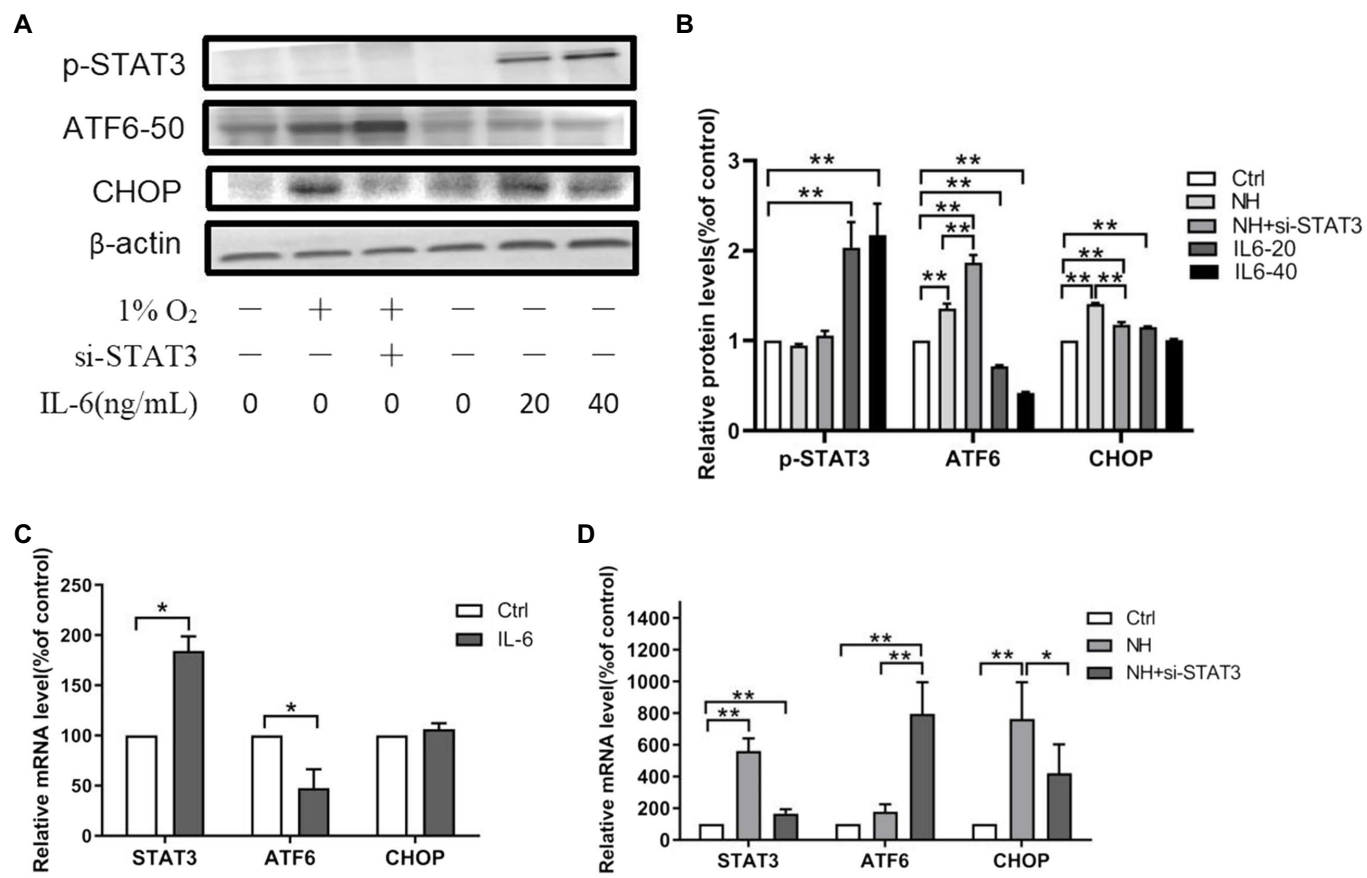
The regulation of STAT3 on ATF6. **(A,B)** The expression of p-STAT3 (Tyr705), ATF6, and CHOP was determined by western blot in PC12, which were incubated with IL-6 or NH+siSTAT3 for 48h. The mRNA levels of STAT3, ATF6, and CHOP were determined by qRT-PCR in PC12, which were incubated with IL-6 **(C)** or NH+siSTAT3 **(D)** for 48h. Data are shown as mean±SD (*n*=3). ^*^p<0.05, ^**^p<0.01 compared with the control group.

The inflammatory factor (IL-6) ligand-receptor interaction causes JAK activation, which in turn activates STAT3 by tyrosine (Tyr705) phosphorylation (Wrighting and Andrews, 2006). Our data showed elevated mRNA of STAT3 in the hypoxia group (Figure 2D). Meanwhile, IL-6 significantly increased the protein levels of p-STAT3 (Tyr705; Figures 2A,B). The above results indicated an enhanced inflammatory response to hypobaric hypoxia in the hippocampus of mice.

### Hypoxia Decreased Viability and Increased Apoptosis of PC12 and HT22 Cells

**Figure 3:**
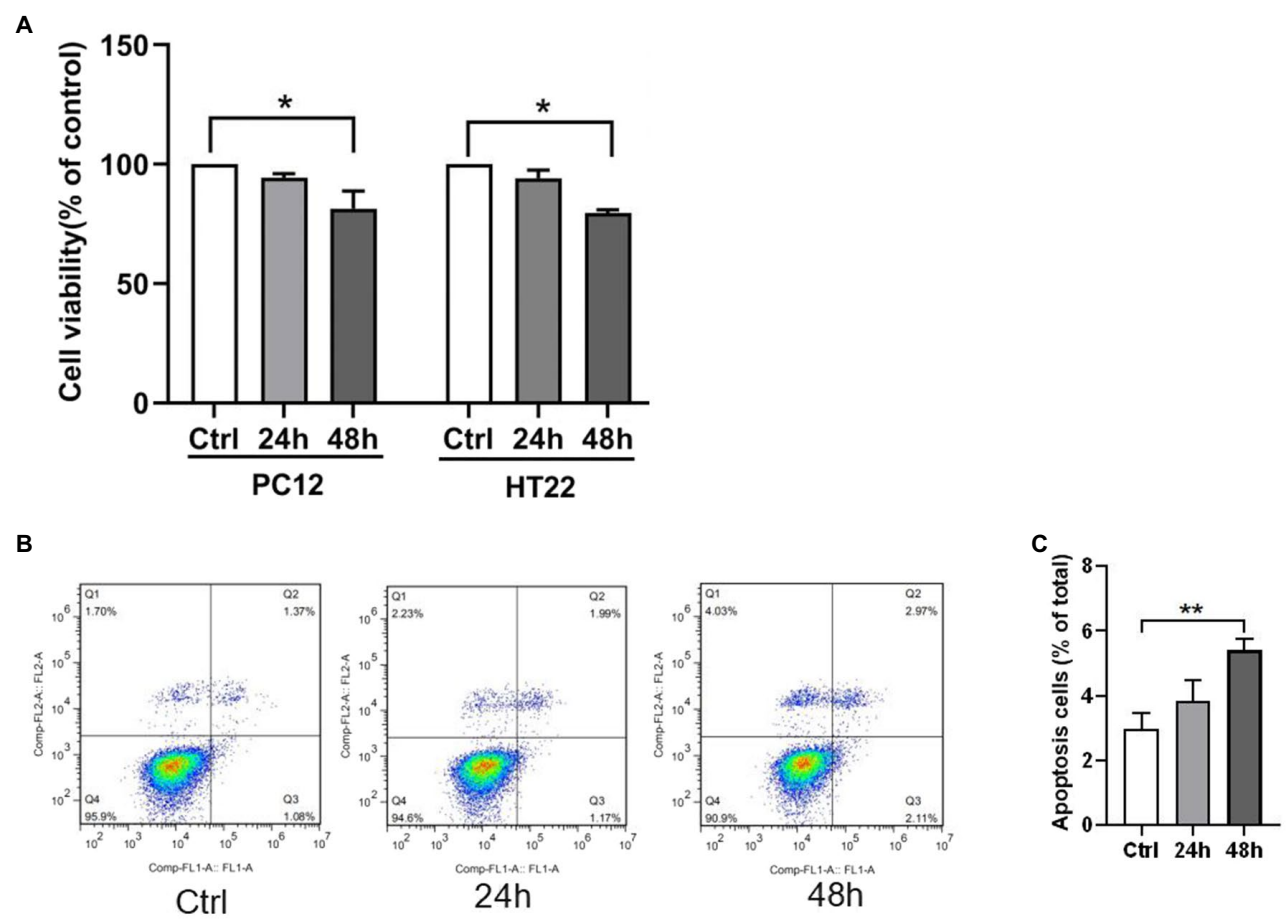
Hypoxia exposure led to elevated neuron apoptosis. **(A)** Cell viability was measured by CCK-8 assay in PC12 and HT22 cells after exposure to 1% O_2_ for 24, 48, and 72h. **(B,C)** The apoptosis of PC12 cells was measured by flow cytometry in PC12 cells after exposure to 1% O_2_ for 24 and 48h. ^*^p<0.05, ^**^p<0.01 compared with the control group.

The CCK8 assay and apoptosis analysis by flow cytometry were performed to investigate the hypoxia effect on the neuron cells’ viability and apoptosis. Exposure to 1% O_2_ for 48h markedly decreased the cell viability (Figure 3A). In apoptosis analysis by flow cytometry, viable cells were not stained by annexin V-FITC or PI (lower left quadrant, Q4), early apoptotic cells were bound with annexin V-FITC (lower right quadrant, Q3), and late apoptotic cells were with V-FITC and PI (upper right quadrant, Q2). Flow cytometry with PI staining and Annexin V displayed a remarkably higher percentage of apoptotic PC12 cell count subjected to 1% O_2_ exposure for 48h than the control group (Figures 3B,C). Among the increased apoptotic cells, the percentage of late apoptosis cells were significantly higher than that of early apoptosis cells (3.58±0.58 vs. 1.82±0.37, *p*<0.05). Therefore, the findings indicate that hypoxia reduced viability and augmented apoptosis in neuron cells.

### Hypoxia Increased ER Stress in Murine Hippocampus and PC12 Cells

**Figure 4:**
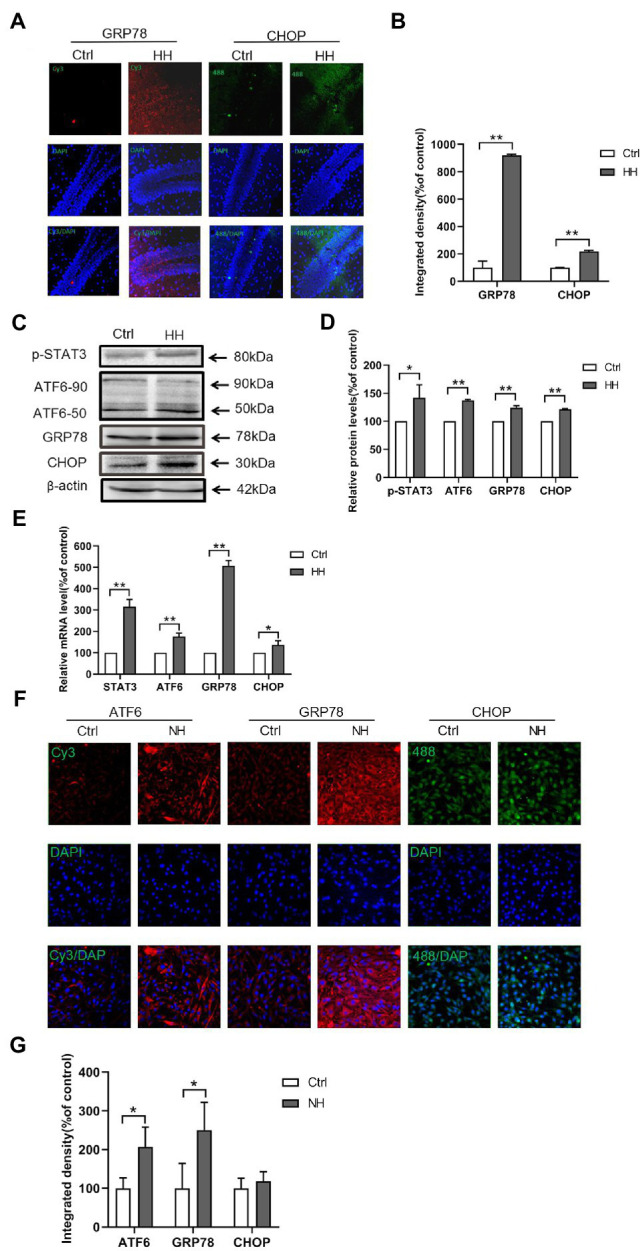
Hypobaric hypoxia (HH) exposure led to the ER stress activation in the hippocampus of C57BL/6. **(A,B)** The expression of GRP78 and CHOP was detected by IF analysis of the frozen sections. **(C,D)** The protein levels of GRP78, ATF6, and CHOP were detected by western blot. **(E)** The mRNA levels of GRP78, ATF6, and CHOP were measured by qRT-PCR. **(F,G)** Normal baric hypoxia (NH) exposure led to the activation of ER stress in PC12 cells. The expression of GRP78, ATF6, and CHOP was detected by IF analysis. ^*^p<0.05, ^**^p<0.01 compared with the control group.

ER stress is significant in HI-induced apoptosis *in vitro* (Chen et al., 2008; Roussel et al., 2013) and *in vivo* (Galehdar et al., 2010; Lopez-Hernandez et al., 2015). The GRP78, ATF6, and CHOP levels were observed in hypoxia animal and cellular models to understand the mechanism of ER stress. The expression of GRP78 and CHOP in the hippocampus showed increased ER stress in the hypobaric hypoxia exposure groups by IF (Figures 4A,B). The proteins related to ER stress, ATF6 and GRP78, and the apoptosis-related protein, CHOP, increased in the hypobaric hypoxia exposure groups (Figures 4C,D). Hypobaric hypoxia mice showed augmented mRNA levels of STAT3, GRP78, ATF6, and CHOP (Figure 4E). The IF staining of ATF6, GRP78, and CHOP in PC12 cells showed increased ER stress in the normal baric hypoxia (NH) exposure groups (Figures 4F,G). Thus, hypoxia increased ER stress *in vivo* and *in vitro*.

### Genome-Wide Characterization of p-STAT3 (Tyr705) Transcriptional Binding Sites in HT22 Cells

**Figure 5:**
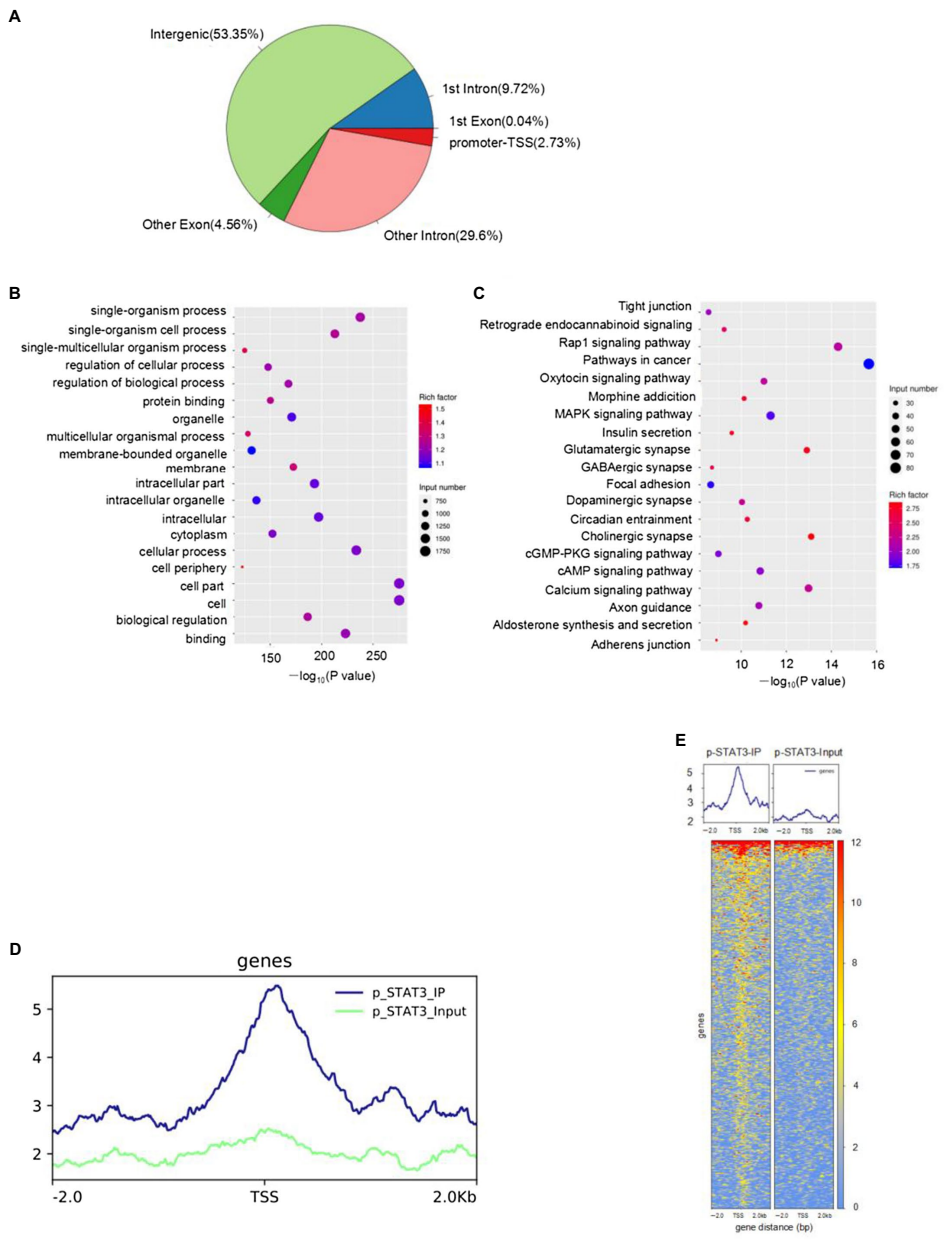
HT22 cells treated with IL-6 were subjected to ChIP-seq with anti-p-STAT3 (Tyr705) antibody. **(A)** Distribution of peak in genome functional regions. **(B)** GO enrichment of peak-related genes (top 20 terms). **(C)** The KEGG enrichment map of metabolic pathways of the peak-related genes (top 20 terms). The bubble color stood for the *p* value, and the bubble size meant the number of genes in the related pathway. **(D,E)** Peak distribution in upstream and downstream TSS.

The STAT3 activation by Tyr705 phosphorylation plays a role in ER stress-induced neuron apoptosis. However, the genomic binding pattern of p-STAT3 (Tyr705) in neurons is not yet clear. We used the ChIP-seq to identify genome-wide target sites of p-STAT3 (Tyr705) in HT22 cells. A total of 2676 p-STAT3 (Tyr705)-enrichment genes were identified. The binding sites located in the promoter TSS made up 2.73% of the total readings of p-STAT3 (Tyr705)-enrichment genes (Figure 5A). The gene ontology (GO) analysis of the genes related to the peak suggested that the p-STAT3 (Tyr705) target genes took part in many biological activities, like single-multicellular organism process, protein binding, and biological regulation (Figure 5B). The Kyoto Encyclopedia of Genes and Genomes (KEGG) analysis implied that the p-STAT3 (Tyr705) genes related to the peak were remarkably enriched in the pathways of metabolism, which included glutamatergic synapse, insulin secretion, dopaminergic synapse, calcium signaling pathway, and cAMP signaling pathway (Figure 5C). From the peak distribution in upstream and downstream transcription start site (TSS), we observed the enrichment of p-STAT3 (Tyr705) binding sites close to TSS (Figures 5D,E), indicating the binding of p-STAT3 (Tyr705) with ATF6 promoter region (Table 2).

**Table 2:** The peak information of ATF6 gene enriched by p-STAT3 using ChIP-seq.

GeneID	Chr	Start	End	Distance to TSS	Fold enrichment	*p*	UniprotAc	Pfam	Interpro
Atf6	1	170,856,069	170,856,356	11,559	3.83	0.00017	Q811K9,Atf6 protein	PF00170,bZIP transcription factor	IPR029801,cAMP-dependent transcription factor ATF-6 alpha; IPR004827, Basic-leucine zipper domain

GeneID, gene name; Chr, the chromosome of peak; Start, start position of peak; End, end position of peak; Distance To TSS, the distance between peak and TSS; Fold enrichment, The enrichment multiple of peak in IP sample vs. input sample; *p* value, The significance *p* value of peak in IP sample vs. input sample; UniprotAc, homologous annotation of ATF6 genes in UniProt database; Pfam, homologous annotation of ATF6 genes in pfam database; Interpro, homologous annotation of ATF6 genes in interpro databaseinterpro.

### IL-6/STAT3 Signaling Affects ATF6 Expression

We examined the ATF6 levels where STAT3 was either overexpressed or silenced in PC12 to ascertain the causal relationship between ATF6 and STAT3 activation. The protein levels and mRNA levels of ATF6 declined in activated p-STAT3 (Tyr705) by IL-6 but upregulated in inhibited STAT3 by siRNA (Figures 2A–D). However, the protein and mRNA expressions of CHOP augmented in activated p-STAT3 (Tyr705) by IL-6 and decreased in inhibited STAT3 by siRNA (Figures 2A–D).

## Discussion

The study demonstrated that the p-STAT3 (Tyr705) signaling by IL-6 was activated in both mice hippocampal and PC12 cells exposed to hypoxia. We studied two ER stress-related genes, ATF6 and GRP78, and apoptosis-related CHOP. The ChIP-seq results showed that ATF6 was significantly enriched by p-STAT3 (Tyr705). Furthermore, we examined the regulatory effects of STAT3 on ATF6, which may be a potential target for reducing neuron injury under hypoxia.

Under the circumstances of hypoxia, the cellular mitochondrial respiration is unsteady producing a large number of free radicals that cause oxidative damage and activate inflammatory reaction (Hartmann et al., 2000; Eltzschig and Carmeliet, 2011; Mukandala et al., 2016). During hypoxia, neurons, microglia, and astrocytes in the brain secrete inflammatory factors (Mukandala et al., 2016). Our previous research has demonstrated that the inflammatory response caused by hypobaric hypoxia induces apparent brain injury (Li et al., 2012). This study examined IL-1β, IL-6, and IL-10 in the mice hippocampal exposed to hypobaric hypoxia for 7d. IL-1β and IL-6 emerged as pro-inflammatory, while IL-10 were an anti-inflammatory and pro-resolution cytokine. We observed significantly higher IL-6 expression and lower IL-10 expression in the hypoxia group than the control group exposed for 7d. Our results suggest the occurrence of the hippocampal inflammatory response in the early hypoxia stage.

The IL-6 ligand-receptor interaction induces JAK activation, which in turn activates STAT3 (Tyr705) phosphorylation (Wrighting and Andrews, 2006). Hypoxia induces STAT3 activation through p-STAT3 (Tyr705; Gao et al., 2015). This study also reported activated p-STAT3 (Tyr705) in the hippocampal slices of the mice brain and PC12. Under hypoxia conditions, STAT3 showed a protective effect in myocardial tissue while showing an injury effect in the brain (Wang et al., 2014; D’Angelo et al., 2016; Hristova et al., 2016). Additionally, we observed that STAT3 was either silenced or overexpressed in PC12 cells. The results also showed that the STAT3 activation promoted CHOP expression, mediating apoptosis in ER stress. However, the downstream mechanism of STAT3-mediated neuronal injury is still not clear.

This study determined the p-STAT3 (Tyr705) downstream by ChIP-seq. Based on the analysis of p-STAT3 (Tyr705) occupation on the whole genome, we proved that p-STAT3 (Tyr705)-enrichment genes play a part in many critical signaling pathways, such as protein binding, organelle, and biological regulation binding. Additionally, highly enriched genes participated in neurotransmitter synthesis signaling pathways involved in dopaminergic synapse, GABAergic synapse, glutamatergic synapse, and cholinergic synapse. Among the various metabolic pathways enriched by p-STAT3 (Tyr705), glutamatergic synapse, insulin secretion, dopaminergic synapse, calcium signaling pathway, and cAMP signaling pathway are related to cognitive function. However, the role of ATF6 in these pathways in the brain is rarely reported. Atf6α−/− mice showed decreased insulin secretion, and ATF6 affected body glucose metabolic homeostasis in specific tissues (Usui et al., 2012). ATF6 has a protective effect on neurotoxin-induced dopaminergic neuron death (Egawa et al., 2011). Our previous research showed that the levels of miR-199a-5p *via* activated STAT3 pathway could be downregulated by chronic hypoxia, and reducing miR-199a-5p facilitates the GRP78 and ATF6 expression in cardiomyocytes (Zhou et al., 2019). The present study focused on the binding of p-STAT3 (Tyr705) to ER stress-related gene, ATF6. The ChIP-seq results showed that p-STAT3 (Tyr705) protein directly binds with ATF6 promoter, suggesting that STAT3 has a direct regulatory effect on ATF6 in neurons, thus explaining the upstream mechanism of ER stress mediated neuron apoptosis. Currently, very few studies are available on the regulatory effect of STAT3 on ATF6. Meng et al. (2020) demonstrated that STAT3 promoted ATF6 transcription, augmenting cellular autophagy by inducing ER stress. These results may be used to explain the transcriptional activity of STAT3 and ATF6 in different tissues.

Hypoxia or ischemia increases the brain’s ER stress. Excessive ER stress increases brain injury, such as neuron apoptosis and blood-brain barrier (BBB) disruption (Baumann et al., 2019; Kam et al., 2019). During hypoxia, the role of ER stress-induced ATF6 in mediated cell survival and apoptosis is still controversial. Some scholars believe that the activation of ATF6 has a protective effect on hippocampal neurons, while others believe that the activation of ATF6 has damaging effects on hippocampal neurons (Pan et al., 2012; Bickler et al., 2015; Jeong et al., 2015; Liu et al., 2015). In this study, we found that hypoxia reduced neuronal cell viability and increased apoptosis; we also explored the ER stress mechanism of hypoxia-induced apoptosis. The study first identified the relationship between STAT3 and ER stress-induced ATF6; when STAT3 was either overexpressed or silenced, the ATF6 levels and CHOP expressions either decreased or increased. The results suggested that activated STAT3 inhibited the expression of ATF6 and upregulated CHOP levels. The findings implied an injury role for STAT3 in hypoxia-induced neuron apoptosis, while ATF6 may have a protective effect.

### Limitations

Although this study confirmed the injury effect of STAT3 on neuron apoptosis through downregulated ATF6 under hypoxia in cells, there are some limitations, such as the application of gene knockout mice. Further research on the cognitive impairment of STAT3 should be verified in animal behavior assays.

## Conclusion

This study suggested that hypobaric hypoxia-induced inflammatory reactions and ER stress in the hippocampus. Additionally, 1% hypoxia exposure augmented p-STAT3 (Tyr705), GRP78, ATF6, CHOP, and elevated the apoptosis ratio in PC12 cells. STAT3 induced neuron injury under hypobaric hypoxia. Overexpressing STAT3 efficiently inhibited ATF6 expression in neurons and might increase hypoxia-induced apoptosis. The p-STAT3 (Tyr705) protein combined with the ATF6 promoter region. In summary, the results suggest that STAT3 has an injury effect on neuron apoptosis through downregulated ATF6 and that it may be a potential target for neuron apoptosis induced by hypoxia.

## Data Availability Statement

The data sets presented in this study can be found in online repositories. The names of the repository/repositories and accession number(s) can be found at: NCBI SRA BioProject, accession no: PRJNA757943.

## Author Contributions

SZ and ZZ carried out cell experiments, animal assays, WB assays, and IF assays. BX carried out animal assays and ELISA experiments. XL and HD conducted WB assays and qRT-PCR assays. GZ participated in the design of the study. YW performed the statistical analysis. PL conceived the study, participated in its design and coordination, and drafted the manuscript along with SZ and PH. All authors contributed to the article and approved the submitted version.

## Funding

This study was supported by grants from the National Natural Science Foundation of China (NSFC; No. 81701854).

## Conflict of Interest

The authors declare that the research was conducted in the absence of any commercial or financial relationships that could be construed as a potential conflict of interest.

## Publisher’s Note

All claims expressed in this article are solely those of the authors and do not necessarily represent those of their affiliated organizations, or those of the publisher, the editors and the reviewers. Any product that may be evaluated in this article, or claim that may be made by its manufacturer, is not guaranteed or endorsed by the publisher.
